# Effect of 50% ethanolic extract of *Syzygium aromaticum *(L.) Merr. & Perry. (clove) on sexual behaviour of normal male rats

**DOI:** 10.1186/1472-6882-4-17

**Published:** 2004-11-05

**Authors:** Tajuddin, Shamshad Ahmad, Abdul Latif, Iqbal Ahmad Qasmi

**Affiliations:** 1 Department of Ilmul Advia (Unani Pharmacology), Faculty of Unani Medicine, Aligarh Muslim University, Aligarh-202002, India

## Abstract

**Background:**

The flower bud of *Syzygium aromaticum *(L.) Merr. & Perry. (clove) has been used in Unani medicine since ancient times for the treatment of male sexual disorders. The present study is aimed to investigate the effect of 50% ethanolic extract of clove on general mating behaviour, libido, potency along with its likely gastric ulceration and adverse effects on sexually normal male albino rats.

**Methods:**

The suspension of the extract was administered orally at the dose of 100, 250, and 500 mg / kg, to different groups of male rats (n = 6) once a day for seven days. The female albino rats involved in mating were made receptive by hormonal treatment. The general mating behaviour, libido and potency were determined and compared with the standard reference drug sildenafil citrate. The probable gastric ulceration and adverse effects of the extract were also evaluated.

**Results:**

Oral administration of the extract significantly increased the Mounting Frequency, Intromission Frequency; Intromission Latency, Erections; Quick Flips, Long Flips as well as aggregate of penile reflexes and caused significant reduction in the Mounting Latency and Post Ejaculatory Interval. The most appreciable effect of the extract was observed at the dose of 500 mg/kg. The test drug was also found to be devoid of any conspicuous gastric ulceration and adverse effects.

**Conclusion:**

The results indicated that the 50% ethanolic extract of clove produced a significant and sustained increase in the sexual activity of normal male rats, without any conspicuous gastric ulceration and adverse effects. Thus, the resultant aphrodisiac effectivity of the extract lends support to the claims for its traditional usage in sexual disorders.

## Background

Clove is the dried flower bud of *Syzygium aromaticum *(L.) Merr. & Perry. (Family: Myrtaceae) an evergreen tree 10–20 m in height indigenous to India, Indonesia, Zanzibar, Mauritius and Sri Lanka [1]. It is one of the most important drugs used in indigenous medicine in India, especially in Unani medicine. Clove is reported as aphrodisiac [2], stomachic [3,4], carminative, antispasmodic [5,6]. It is reported to be useful in conceiving in high doses and act as a contraceptive in low doses [7] and useful in cataract [8]. Clove is also reported to have anticarcinogenic property [9]. It inhibits platelet aggregation and alters arachidonic acid metabolism in human platelets [10]. It posseses antiviral activity against *Herpes simplex *[11]. Phytochemical studies indicate that the clove contains free eugenol, eugenol acetate, caryophyllene, sesquetrepene ester [12], phenyl propanoid [13], β caryophyllene [14], eugenol and acetyle eugenol [15]. Eugenol, the major constituent, inhibits lipid peroxidation and maintains activities of enzyme superoxide dismutase, catalase, glutathione peroxidase-6 phosphate dehydrogenase [16], and has also been reported to have vasodilatory [17] and smooth muscle relaxant property [18]. Phyto chemical study of the test drug that was carried out according to the methods described by Jenkin et al [19], showed that it contains alkaloids, amino acids, flavonoids, proteins, sterols, reducing sugar, tannins and phenols. However, clove, or its known compound had not been scientifically studied for their effect on sexual function. Earlier we carried out a preliminary study of hydro alcoholic extract (50%) of clove using only mounting frequency and mating performance as the marker for sexual function in normal male mice, and the results from our study demonstrated the aphrodisiac activity and safety from short term toxicity of the test drug [20].

The present study thus, is aimed to investigate the aphrodisiac effect of 50% ethanolic extract of clove in detail, using multiple parameters along with its probable gastric ulceration and adverse effects in sexually normal male albino rats. The doses used in the study were selected according to the Freirich [21], multiplying the Unani clinical doses reported in standard Unani text [2] by the conversion factor of 7.

## Methods

### Plant material and extraction

The authenticated dried flower bud of *S. aromaticum *(clove) was procured from the market (Delhi, India). A voucher (S721) sample was kept for further reference. The clove was crushed to coarse powder and sieved through No. 20 mesh size. The extraction was carried out by mixing the powdered clove with 1:3 w/v in 50% ethanol v/v by Soxhlet apparatus for 6 h. The extract was filtered and the solvent from the filtrate was removed by rotary evaporator under reduced pressure and low temperature. The yield of extract was 10.40% w/w in terms of dried starting material. It was yellowish and of pleasant smell. The extract was preserved in a refrigerator.

### Chemicals used

Sildenafil citrate was purchased from Zydus Cadila, (Ahmadabad, India). Other drugs used were ethinyl oestradiol (Infar Limited, Calcutta, India), progesterone (Sun Pharmaceutical Industries Limited, Mumbai, India) and 5% xylocane ointment (Astra IDL Limited, Bangalore, India)

### Animals

Twelve weeks old male and female albino rats of wistar strain weighing 350–400 g and 225–275 g respectively, were used for the study. They were housed singly in separate standard cages and maintained under standard laboratory conditions (temperature 24–28°C, relative humidity 60–70%, 12 h light-dark cycle) with free access to solid pellet diet (Gold Mohar, Lipton-India) and water *ad libitum *throughout the study except during the experiment. The study design was approved by the ethical committee of the Department for animal care and use.

### Drug preparation

Since clove in Unani medicine is orally administered, therefore, the extract of clove was suspended in distilled water using Tween 80 (1%) for oral administration. Sildenafil citrate and ethinyl oestradiol were also suspended in distilled water using Tween 80 (1%) separately, for oral use. Progesterone was dissolved in olive oil for subcutaneous injection. All the drug solutions were prepared just before administration.

### Mating behaviour test

The test was carried out by the methods of Dewsbury and Davis Jr [22] and Szechtman et al [23], modified by Amin et al [24]. Healthy and sexually experienced male albino rats (350–400 g) that were showing brisk sexual activity were selected for the study. They were divided into 5 groups of 6 animals each and kept singly in separate cages during the experiment. Group 1 represented the control group, which received 10 ml/kg of distilled water orally. Groups 2–4 received suspension of the extract of clove orally at the doses of 100, 250 and 500 mg/kg, respectively, daily for 7 days at 18:00 h. Group 5 served as standard and given suspension of sildenafil citrate orally at the dose of 5 mg/kg, 1 h prior to the commencement of the experiment. Since the male animals should not be tested in unfamiliar circumstances the animals were brought to the laboratory and exposed to dim light (in 1 w fluorescent tube in a laboratory of 14' × 14') at the stipulated time of testing daily for 6 days before the experiment.

The female animals were artificially brought into oestrus (heat) [25] by the Szechtman et al method (as the female rats allow mating only during the estrus phase) They were administered suspension of ethinyl oestradiol orally at the dose of 100 μg/animal 48 h prior to the pairing plus progesterone injected subcutaneously, at the dose of 1 mg/animal 6 h before the experiment. The receptivity of the female animals was confirmed before the test by exposing them to male animals, other than the control, test and standard animals. The most receptive females were selected for the study. The experiment was carried out on the 7^th ^day after commencement of the treatment of the male animals. The experiment was conducted at 20:00 h in the same laboratory and under the light of same intensity. The receptive female animals were introduced into the cages of male animals with 1 female to 1 male. The observation for mating behaviour was immediately commenced and continued for first 2 mating series. The test was terminated if the male failed to evince sexual interest. If the female did not show receptivity she was replaced by another artificially warmed female. The occurrence of events and phases of mating were called out to be recorded on audio-cassette as soon as they appeared. Their disappearance was also called out and recorded. Later, the frequencies and phases were determined from cassette transcriptions: number of mounts before ejaculation or Mounting Frequency (MF), number of intromission before ejaculation or Intromission Frequency (IF), time from the introduction of female into the cage of the male upto the first mount or Mounting Latency (ML), time from the introduction of the female up to the first intromission by the male or Intromission Latency (IL), time from the first intromission of a series upto the ejaculation or Ejaculatory Latency (EL), and time from the first ejaculation upto the next intromission by the male or Post Ejaculatory Interval (PEI). In the second mating series only the EL was recorded. The values for the observed parameters of the control, test and standard animals were statistically analysed by using one-way analysis of variance (ANOVA) method.

### Test for libido

The test was carried out by the method of Davidson [26], modified by Amin et al [24]. Sexually experienced male albino rats were divided into 5 groups of 6 animals each and kept singly in separate cages during the experiment. Group 1 represented the control group, which received 10 ml/kg of distilled water orally. Groups 2–4 received suspension of the extract orally at the doses of 100, 250 and 500 mg/kg, respectively, once a day in the evening (18:00 h) for 7 days. Group 5 served as standard and given suspension of sildenafil citrate orally at the dose of 5 mg/kg, 1 h prior to the commencement of the experiment. The female rats were made receptive by hormonal treatment and all the animals were accustomed to the testing condition as previously mentioned in mating behaviour test. The animals were observed for the Mounting Frequency (MF) on the evening of 7^th ^day at 20:00 h. The penis was exposed by retracting the sheath and 5% xylocaine ointment was applied 30, 15 and 5 min before starting observations. Each animal was placed individually in a cage and the receptive female rat was placed in the same cage. The number of mountings was noted. The animals were also observed for intromission and ejaculation. The MF in control, test and standard animals was statistically analysed by employing one-way analysis of variance (ANOVA) method.

### Test for potency

The effect of the test drug was studied according to the methods described by Hart and Haugen [27] and Hart [28], modified by Amin et al [24]. The male animals were divided into 5 groups of 6 animals each and kept singly in separate cages during the experiment. Group 1 represented the control group, which received 10 ml/kg of distilled water orally. Groups 2–4 received suspension of the test drug orally at the doses of 100, 250 and 500 mg/kg, respectively, daily for 7 days. Group 5 received a suspension of sildenafil citrate orally at the dose of 5 mg/kg, 1 h before the commencement of the experiment. On the 8^th ^day, the test for penile reflexes was carried out by placing the animal on its back in a glass cylinder partial restraint. The preputial sheath was pushed behind the glans by means of thumb and index finger and held in this manner for a period of 15 min. Such stimulation elicits a cluster of genital reflexes. The following components were recorded: Erections (E), Quick Flips (QF), and Long Flips (LF). The frequency of these parameters observed in control, test and standard groups was statistically analysed by using one-way analysis of variance (ANOVA) method.

### Test for ulcerogenecity

The male animals (350–400 g) were divided into 4 groups of 6 animals each. Group 1 represented the control group, which received 10 ml/kg of distilled water. Groups 2–3 received suspension of the extract orally at the doses of 100, 250 and 500 mg/kg, respectively, daily for 7 days. After the treatment, on 8^th ^day all the animals were killed and the stomach was then incised along the greater curvature and washed carefully with physiological saline. Any gastric lesions were observed immediately using a magnifying glass. The number of erosions per stomach was assessed for severity, according to the score of Cioli et al [29] : (o) absence of lesion, vasodilation or up to 3 pin point ulcers; (1) more than 3 pin point ulcers, (2) from 1 to 5 small ulcers (< 2 mm); (3) more than 5 small ulcers (< 2 mm), (4) 1 or more giant ulcers. Evaluation of gastric damage was carried out by two observers, who followed the same evaluation criteria.

### Adverse effects

All treated rats were observed at least once daily for any overt sign of toxicity (salivation, rhinorrhoea, lachrymation, ptosis, writhing, convulsions and tremors), stress (erection of fur and exophalmia) and changes in behaviour (such as spontaneous movement in the cage, climbing, cleaning of face). In addition food and water intake were noted.

### Statistical analysis

The significance of difference between the means was determined by one-way analysis of variance (ANOVA) with post-hoc't' test. P value <0.05 was considered as significant.

## Results

The data obtained with the mating behaviour test indicated that the clove-extract at the dose of 500 mg/kg increased the Mounting Frequency (MF) (P < 0.0001), Intromission Frequency (IF) (P < 0.001) ; Ejaculatory latency in first series (EL_1_) (P < 0.0001) and decreased Mounting Latency (ML) (P < 0.0001), Intromission Latency (IL) (P < 0.01) in a significant manner. The dose 250 mg / kg of the extract significantly increased the MF (P < 0.001), IF (P < 0.05) and significantly reduced the ML (P < 0.001), EL_1 _(P < 0.05); Post Ejaculatory Interval (PEI) (P < 0.001) and did not significantly alter the IL (P < 0.61), Ejaculatory Latency in second series (EL_2_) (P < 0.41). Whereas the dose of the test drug at 100 mg/kg significantly increased the MF (P < 0.05), PEI (P < 0.05) but did not significantly affect the IF (P < 0.61), ML (P < 0.61), IL (P < 0.61); EL_1 _(P < 0.61); EL_2 _(P < 0.81). However, the standard drug increased the MF (P < 0.0001), IF (P < 0.0001); EL_1 _(P < 0.0001), EL_2 _(P < 0.0001); PEI (P < 0.0001) as well as decreased ML ((P < 0.0001) and IL (P < 0.0001) in a highly significant manner (Table 1).

**Table 1:** Effect of 50% ethanolic extract of clove (*S. aromaticum*) on mating behaviour in male rats

	Mean Frequency ± SEM				
Parameters	Control (10 ml/kg)	Clove (100 mg/kg)	Clove (250 mg/kg)	Clove (500 mg/kg)	Sildenafil citrate (5 mg/kg)

Mounting Frequency (MF)	11.50 ± 1.22	13.20 ± 1.47*	19.00 ± 1.10***	31.80 ± 2.32****	48.70 ± 2.34****
Intromission Frequency (IF)	5.50 ± 1.22	5.83 ± 1.17 NS	6.67 ± 0.81*	8.17 ± 1.47***	24.70 ± 0.81****
Mounting Latency (ML, in sec)	35.30 ± 1.51	36.70 ± 1.21 NS	31.30 ± 1.63***	24.30 ± 1.63****	11.70 ± 1.37****
Intromission Latency (IL, in sec)	40.00 ± 5.29	41.30 ± 3.89 NS	38.50 ± 3.89 NS	21.00 ± 3.83**	15.00 ± 0.89****
Ejaculatory Latency in first series (EL_1, _in sec)	198.00 ± 0.98	201.00 ± 13.00 NS	208.00 ± 13.70*	233.50 ± 12.00****	344.50 ± 12.00****
Ejaculatory Latency in second series (EL_2_, in sec)	297.33 ± 8.10	295.50 ± 11.70 NS	311.33 ± 9.67 NS	343.16 ± 7.70****	398.16 ± 13.50****
Post Ejaculatory Interval (PEI, in sec)	364.00 ± 12.22	343.16 ± 7.70*	309.33 ± 10.90****	217.33 ± 8.96****	99.00 ± 5.68****

Tabular values are mean ± SEM, n = 6 (number of animals in each group); significant difference from control, NS: Not significant. * P < 0.05, ** P < 0.01; *** P < 0.001, **** P < 0.0001.

The test for libido showed that the extract at the dose of 500 mg/kg increased the Mounting Frequency (MF) in a significant manner (P < 0.001). The extract at the doses of 100 mg/kg and 250 mg/kg did not significantly alter the MF(P < 0.24, P < 0.75 respectively). The standard drug strikingly increased the MF (P < 0.0001). Intromission and Ejaculation were found absent in control, test and standard groups (Table 2).

**Table 2:** Effect of 50% ethanolic extract of clove (*S. aromaticum*) on Mounting Frequency (test for libido) in male rats

	Mean Frequency ± SEM				
	
Parameters	Control (10 ml/kg)	Clove (100 mg/kg)	Clove (250 mg/kg)	Clove (500 mg/kg)	Sildenafil citrate (5 mg/kg)
Mounting Frequency (MF)	6.17 ± 0.98	6.33 ± 0.81NS	7.17 ± 1.72 NS	11.20 ± 1.17*	23.00 ± 2.17**
Intromission Frequency (IF)	Nil	Nil	Nil	Nil	Nil
Ejaculation (EJ)	Absent	Absent	Absent	Absent	Absent

Tabular values are mean ± SEM, n = 6 n (number of animals in each group); significant difference from control, NS: Not significant. *P < 0.001,** P < 0.0001

The test for potency exhibited that the higher dose (500 mg/kg) of the test drug significantly increased the frequency of Erections (E) (P < 0.0001), Quick Flips (QF) (P < 0.0001), Long Flips (LF) (P < 0.0001) as well as the aggregate of these penile reflexes (TPR) (P < 0.0001). The extract at the dose of 250 mg/kg significantly increased the E (P < 0.01), LF (P < 0.05) and TPR (P < 0.05) but comparatively less than the higher dose of the extract and standard drug, and did not significantly increase the QF (P < 0.10). whereas, the test drug at the dose of 100 mg/kg did not alter the E (P < 0.78), QF (P < 0.78); LF (P < 0.44) and TPR (P < 0.44) in a significant manner (Table 3).

**Table 3:** Effect of 50% ethanolic extract of clove (*S. aromaticum*) on Penile reflexes (test for potency)

	Mean Frequency ± SEM				
	
Parameters	Control (10 ml/kg)	Clove (100 mg/kg)	Clove (250 mg/kg)	Clove (500 mg/kg)	Sildenafil citrate (5 mg/kg)
Erections (E)	7.67 ± 1.63	8.33 ± 1.21 NS	10.50 ± 1.76**	13.80 ± 0.98***	19.00 ± 2.64***
Quick Flips (QF)	5.17 ± 0.75	5.33 ± 1.21 NS	6.00 ± 1.22 NS	9.67 ± 1.37***	17.30 ± 4.13***
Long Flips (LF)	2.17 ± 1.17	3.17 ± 1.50 NS	4.00 ± 1.55*	7.50 ± 1.22***	12.00 ± 2.26***
Total Penile Reflexes (TPR)	15.01 ± 3.55	16.83 ± 7.21 NS	20.50 ± 4.53*	30.97 ± 3.50***	48.30 ± 9.03***

Tabular values are mean ± SEM, n = 6 (number of animals in each group); significant difference from control, NS: Not significant. * P < 0.05, ** P < 0.01; *** P < 0.0001.

Seven days treatment of low and high doses of the extract caused no significant ulceration in gastric mucosa of albino rats. Moreover, there were neither treatment related defects nor overt clinical signs of toxicity, stress or changes in behaviour and appearance evident. The food and water intake of all test drug treated rats remained similar to those of the control group.

## Discussion

In the present study, clove (*S. aromaticum*) was tested in animal experimentation for its effect on sexual behaviour, and sildenafil citrate was used as the standard referent.

The study showed that the 50% ethanolic extract of clove possesses significant sexual function enhancing activity as observed in sexual behaviour tests. Mating behaviour test revealed that the test drug significantly increased the Mounting Frequency (MF) and Intromission Frequency (IF) as compared to control but less than that of the standard drug. The (MF) and (IF) are considered as the indices of both libido and potency. So, this is an indication that the test drug possesses a sexual function improving effect. The premature ejaculation is one of the important causes of sexual dysfunction, so the assessment of Ejaculatory Latency in first series (EL_1_) and in second series (EL_2_) was studied. The test drug significantly increased the EL_1 _and EL_2 _as compared to control animals, whereas a highly significant increase was observed with the standard drug.

The test drug was found to produce a significant reduction in the Mounting Latency (ML) and Intromission Latency (IL) as compared to control while a highly significant decrease was found in ML of animals treated with sildenafil citrate. This is also an evidence of the sexual function improving effect of the clove.

The Post Ejaculatory Interval (PEI) is considered as an index of potency and libido, and also a parameter of the rate of recovery from exhaustion after first series of mating. PEI was found significantly decreased with clove-extract and also with the standard drug. The test drug decreased PEI either by enhancing the potency and libido or by producing lesser exhaustion in the first series of mating or by both.

The effect of the test drug on libido was also evaluated by testing the MF after genital anaesthetization which does away with the reinforcing effect of genital sensation thus affording the study of pure libido or intrinsic sexual desire. During the experiment the extract produced a significant increase in the MF of sexually normal male rats whereas the efficacy of the standard drug, as expected, was found to be highly significant. The MF was much reduced in control, test and standard animals in comparison with the MF of corresponding groups in mating behaviour test where the penis had not been anaesthetized. None of the control, test and standard animals were observed to show Intromission or Ejaculation because their occurrence depends upon local genital sensation which was obstructed due to anaesthetization.

The effect on potency was also evaluated by testing the effect of the drug on the frequency of penile reflexes namely Erections (E), Quick Flips (QF), and Long Flips (LF). The test drug significantly increased the frequency of all the components of penile reflexes (E, QF, & LF) in the test animals as compared to control group but comparatively lesser than the standard drug. The aggregate of penile reflexes (TPR) was also found increased in the animals treated with the extract and sildenafil citrate. Therefore, the experiment revealed that the test drug produced a marked increase in potency.

The spices are reported to produce an increase in gastric acid secretion by a cholinergic mechanism [30], and so, their use for sexual invigoration may cause gastric ulceration and other adverse effects. Therefore, ulcerogenic and other adverse effects of the extract were also evaluated. The results of this evaluation were negative. This suggests that the short term use of clove for this purpose is apparently safe.

The results of the present study clearly proved that the clove is endowed with sexual function improving activity. This is in consonance with our earlier study showing sexual function improving effect of the test drug in male mice. However, the established drug i.e. sildenafil citrate exhibited, as expected, tremendous activity. With regard to the mechanism of the test drug, it is difficult to interprete the mechanism involved in potentiation of sexual function. The drugs induced changes in neurotransmitter levels or their action at cellular levels could change sexual behaviour [31]. Hence, the increased sexual function could be due to the nervous stimulant action of the test drug [32]. Further, phyto chemical study of the extract indicated that it contains sterols and phenol. Thus, the resultant aphrodisiac effectivity of the test drug might also be attributed to sterols or phenolic compounds.

Moreover, research should be aimed at isolating the active principle(s) responsible for aphrodisiac activity and the mechanism by which the drug enhances sexual function. In addition, to discover the applied effective concentration or dosages of the extract, more studies are also required.

## Conclusions

The present results indicated that the 50% ethanolic extract of clove possesses potent aphrodisiac activity in normal male albino rats without any gastric ulceration and adverse effects and provided scientific evidence in favour of the claims made in Unani medicine that the clove is clinically useful as sexual invigorator in males.

## Competing interests

The author(s) declare that they have no competing interests.

## Authors' contributions

T-Supervised the design and coordinatrion of the study.

SA-Practically conducted the design of the study.

AL-Participated and performed the statistical analysis

IA-Participated in the drafting of manuscript.

All authors read and approved the final manuscript.

## Pre-publication history

The pre-publication history for this paper can be accessed here:



## References

[B1] Trease GE, Evans WC, Trease GE, Evans WC  (1972). *Eugenia caryophyllata*. A Text book of pharmacognosy.

[B2] Khan MA, Khan MA (1893). Qaranful (clove). Moheet-e-Azam (Persian).

[B3] Nadkarni KM, Nadkarni AK (2000). *Myrtus caryophyllus*. Materia Medica.

[B4] Attar HZ, Attar HZ (1370). Qaranful (clove). Ikhtiyarat-e-Badiyee (Persian).

[B5] Khory RN, Katrak NN, Khory RN, Katrak NN (1985). *Eugenia caryophyllata*. Materia Medica of India and their therapeutics.

[B6] Pourgholami MH, Kamalinejad M, Javadi M, Majzoob S, Sayyah M (1999). Evaluation of the anticonvulsant activity of the essential oil of *Eugenia caryophyllata *in male mice. J Ethnopharmacol.

[B7] Baytar Ibn, Baytar Ibn (1869). Qaranful (clove). Al Jame' Li-Mufradat al Advia wal Aghzia (Arabic).

[B8] Raazi ABM, Raazi ABM (1961). Qaranful (clove). In : Kitab al Haawi Fi al Tibb (Arabic).

[B9] Zheng GQ, Kenney PM (1992). Sesqueterpenes from clove (*Eugenia caryophyllata*) as potential anti carcinogenic agents. J Nat Prod.

[B10] Srivastava KC, Malhotra N (1991). Acetyl eugenol, a component of oil of cloves (*S. aromaticum*) inhibits aggregation and alters arachidonic acid metabolism in human blood platelets. Prostaglandins Leukot Essent Fatty Acids.

[B11] Kurkawa M, Hozumi T, Basnet P, Nakano M, Kedota S, Namba T, Kawana T, Shiraki K (1998). Purification and characterization of eugeniine as anti herpes virus compound from *Geum japanicum *and *Syzygium aromaticum*. J Pharmacol Exp Ther.

[B12] Rastogi RP, Mehrotra BN, Rastogi RP, Mehrotra BN (1984). *Syzygium aromaticum*. Compendium of Indian Medicinal Plants.

[B13] Miyazawa M, Hisama M (2003). Antimutagenic activity of Phenylpropanoids from clove (*Syzygium aromaticum*). J Agric Food chem.

[B14] Ghelardini C, Galeotti N, Di Cesare Mannelli L, Mazzanti G, Bartolini A (2001). Local anaesthetic activity of beta-caryophyllene. Farmaco.

[B15] Srivastava KC (1993). Antiplatelets principles from a food spice clove (*Syzygium aromaticum *L.). Prostaglandins Leukot Essent Fatty Acids.

[B16] Kumarvelu P, Subramanyam S, Dakshin murthy DP, Devraj NS, Kumarvelu P (1996). The antioxident effect of eugenol on carbon tetrachloride-induced erythrocyte damage in rats. Nut Biochem.

[B17] Criddle DN, Madeira SV, Soares de Moura R (2003). Endothelium – dependent and – independent vasodilator effects of eugenol in the rat mesentric vascular bed. J Pharm Pharmacol.

[B18] Damiani CE, Rossoni LV, Vassallo DV (2003). Vasorelaxant effect of eugenol on rat thorasic aorta. Vascul Pharmacol.

[B19] Jenkins GL, Knevel AM, Digangi FE (1967). Quantitative pharmaceutical chemistry.

[B20] Tajuddin, Ahmad S, Latif A, Qasmi IA (2003). Aphrodisiac activity of 50% ethanolic extracts of *Myristica fragrans *Houtt. (nutmeg) and *Syzygium aromaticum *(L.) Merr. & Perry. (clove) in male mice: a comparative study. BMC complement Altern Med.

[B21] Freirich EJ (1968). Quantitative comparison of toxicity of anti-cancer agents in mouse, rat, dog, monkey and man. Cancer Chemotherapy Report.

[B22] Dewsbury DA, Davis HN (1970). effect of Reserpine on the copulatory behaviour of male rats. Physiol Behav.

[B23] Szechtman H, Moshe H, Rabi S (1981). Sexual behaviour Pain sensitivity and stimulates endogenous opioid in male rats. Eur J Pharmacol.

[B24] Amin KMY, Khan MN, Rahman SZ, Khan NA (1996). Sexual function improving effect of *Mucuna pruriens *in sexually normal male rats. Fitoterapia.

[B25] Ratna Sooriya WD, Dharmasiri MG (2000). Effect of *Terminalia catappa *seeds on sexual behaviour and fertility of male rats. Asian J Androl.

[B26] Davidson JM, Zewi H (1982). Sexology, sexual biology, behaviour and therapy: selected papers of Fifth World Congress of sexology Jerusalem 1981.

[B27] Hart BL, Haugen CM (1968). Activation of sexual reflexes in male rats by spinal implementation of testosterone. Physiol Behav.

[B28] Hart BL (1979). Activation of sexual reflexes in male rats by dihydrotestosterone but not estrogen. Physiol Behav.

[B29] Cioli V, Silvestrini B, Dordoni F (1967). Evaluation of potential of gastric ulceration after administration of certain drugs. Exp Mol Pathol.

[B30] Vasudevan K, Vembar S, Veeraraghavan K, Haranath PS (2000). Influence of intragastric perfusion of aqueous spice extracts on acid secretion in anaesthetized albino rats. Indian J Gastroenterol.

[B31] Suresh Kumar PK, Subramoniam A, Pushpangadan P (2000). Aphrodiasiac activity of *Vanda tessellata *(ROXB.) HOOK. EXDON. extract in male mice. Indian J Pharmacol.

[B32] Sina Ibn, Sina Ibn (1906). Qaranful (clove). Al Qanoon Fi al Tibb (Arabic).

